# Cross-Sectional Analysis of the Relationship Between Adherence to the Mediterranean Diet and Mental Wellness

**DOI:** 10.7759/cureus.34878

**Published:** 2023-02-11

**Authors:** Gabrielle A Jasmin, Kellie N Fusco, Stephanie N Petrosky

**Affiliations:** 1 Nutrition, Nova Southeastern University Dr. Kiran C. Patel College of Osteopathic Medicine, Fort Lauderdale, USA; 2 Osteopathic Medicine, Nova Southeastern University Dr. Kiran C. Patel College of Osteopathic Medicine, Fort Lauderdale, USA

**Keywords:** depression, anxiety, diet, mental well-being, mediterranean diet

## Abstract

The purpose of the study was to determine whether there was a relationship between adherence to the Mediterranean diet (MD) and levels of anxiety, depression, and overall mental well-being. The Mediterranean diet is a popular, healthy diet, aimed to promote wellness and reduce chronic illness. In order to determine the relationship between MD and mental well-being, 100 participants consented to complete an online survey to analyze their adherence to MD, along with levels of anxiety and depression. The validated questionnaires of the 14-item Questionnaire of Mediterranean diet Adherence, Generalized Anxiety Disorder-7 (GAD-7), and Beck's Depression Inventory (BDI) assessments were used to analyze each participant. To evaluate the results of the study, Spearman's rank correlation coefficient analysis was used to identify relationships between MD, depression, and anxiety. There was a significant negative correlation, indicating that MD adherence is associated with reduced depression and anxiety.

## Introduction

First described by Ancel Keys in the 1960s, the Mediterranean diet (MD) is a dietary pattern consisting of healthy foods such as fruits, vegetables, whole grains, legumes, fish, and nuts [1]. Rich in fiber, antioxidants, and omega-3 fatty acids, MD limits consumables such as processed foods, red meats, refined grains, and added sugars [1]. Research demonstrates the benefits of MD, such as preventing cardiovascular disease and reducing the risks associated with diabetes, hypertension, and obesity [1-3]. The Mediterranean diet is also useful in improving insulin resistance (IR) [3].

Mental wellness

Growing evidence has shown that partaking in the MD can be beneficial to not only physical health but mental health as well [1]. One group of researchers [4] analyzed the emotional well-being of university students using two different assessments. A 14-point questionnaire on adherence to the MD was given to the students along with a questionnaire measuring various positive and negative moods. The authors determined a significant inverse relationship between MD and emotional well-being. The authors also suggested that the high intake of key components of MD, such as olive oil, fish, fruits, vegetables, and legumes, particularly contributed to the reported lower levels of anxiety and depression. Overall, adherence to MD was positively associated with a more positive outlook and quality of life.

Adherence to MD has been shown to reduce the severity of depressive symptoms in adults living in the United States. In 2019, the National Institute of Mental Health Information Resource Center reported that 19.4 million adults, or 7% of U.S. adults, experienced a major depressive episode [5]. Depression has been shown to be more prevalent in women than men, potentially due to hormonal shifts during puberty, menstruation, pregnancy, and menopause [6]. Depression is also a leading cause of disability with pharmaceutical mechanisms often producing ineffective results or related side effects [5]. An anti-inflammatory diet, such as MD, can prevent or reduce the symptoms of depression [5]. Others have studied the effects of MD adherence on feelings of depression, collecting detailed information about participants' diets using a food frequency questionnaire and other variable factors such as body mass index, level of physical activity, and smoking use. Data collected from 49,261 Swedish women found that adherence to MD was negatively correlated with lower levels of depression [7]. Since depression is so prevalent in the United States [5], more evidence is needed to validate the benefits of using MD to enhance overall well-being.

Despite MD being recommended to the general population, there are known discrepancies in adhering to it, especially between different racial/ethnic groups. One study indicated that positive effects, such as cardiovascular disease benefits, were achieved for only individuals at higher socioeconomic levels [8]. This possibly indicates that the MD may not be available to certain racial/ethnic groups with well-documented socioeconomic disparities and poor food security. Despite this affordability concern, it has been shown that such populations may still be able to adhere to Mediterranean-like foods that are both culturally appropriate and cost-effective. Such foods include beans, canned tuna, and frozen or canned fruits and vegetables [9-10]. These concerns should be kept in consideration when assessing adherence to the MD across all racial/ethnic groups. The well-being of the general public regarding nutrition should be of high priority to healthcare professionals, especially to the most vulnerable groups due to their risk of significant health disparities and disproportionally high multimorbidity [10].

Patients and healthcare professionals need to consider diet when evaluating one’s lifestyle and well-being [11]. The use of self-reflective tools in treatment plans can be enlightening and engage patients in their care. Patients can also report foods that trigger symptoms or unpleasant effects, while also reporting which foods produce "feel good" effects. Various nutritional deficiencies, including zinc and magnesium, can also play a role in a low mood [12]. Since MD encourages the consumption of multiple food groups, people who adhere to it often consume a nutrient-dense diet, while also being at a reduced risk of developing a nutritional deficiency, compared to those who consume a Western diet [13]. The aim of this study was to further document the relationship of MD adherence with self-reported levels of anxiety and depression. Combined in a unique way, we hypothesized that higher adherence to MD would be correlated with lower levels of anxiety and depression.

## Materials and methods

Institutional Review Board

The survey research study was submitted for approval to the Institutional Review Board (IRB) at Nova Southeastern University through the Dr. Kiran C. Patel College of Osteopathic Medicine. The study was approved on June 20, 2022, with the IRB number 2022-277.

Recruitment

Participants over the age of 18 were eligible to take part in the survey. Recruitment of participants was conducted by marketing the survey on bulletins at fitness clubs in South Florida and globally on the social media platforms Facebook and Instagram. The marketing flier contained the survey's website link and a quick response (QR) code that could be paired with a mobile device.

The inclusion criteria required participants to: 1) be at least 18 years old, 2) be proficient in the English language, 3) have access to a computer to participate in the electronic survey or a mobile device to scan a quick response code, and 4) give implied consent.

The exclusion criteria prohibited participants who: 1) were under the age of 18 years, 2) were not proficient in the English language, 3) did not have access to an electronic device, or 4) did not give implied consent.

Instruments

Participants were first presented with the online Informed Consent Form. If consent was given, participants selected the ‘I agree’ button which brought them to the beginning of the survey. The online survey consisted of four separate sections: Demographics, the 14-item Questionnaire of Mediterranean Diet Adherence, as shown in Figure 3 [14-17], Generalized Anxiety Disorder-7 (GAD-7) [18], and Beck’s Depression Inventory (BDI) [19]. The entire survey took approximately 10 minutes to complete. 

Demographic information was obtained following informed consent. The data collected in this section included age, gender identity, and race/ethnicity.

The 14-item Questionnaire of Mediterranean Diet Adherence is a validated questionnaire that consists of 14 items used to measure adherence to MD [14]. Questions involve inquiring about the participants' typical dietary choices. Each item is associated with one point and is obtained if the criteria for each question are met (as shown in Figure 3 in the Appendices). Higher scores indicate higher adherence to MD [14].

GAD-7 is a commonly used self-reported questionnaire that consists of seven items to measure the severity of anxiety. It is utilized as an initial screening tool for generalized anxiety disorder [18]. In each item, GAD-7 asks participants to rate how often they experience a specific feeling related to a symptom of anxiety over the last two weeks, with the frequency options of "not at all", "several days", "more than half the days", and "nearly every day." The items are measured on a 3-point scale, ranging from 0 (not at all) to 3 (nearly every day). The score of each item is then combined to calculate the GAD-7 total score, which can range from 0 to 21. A score of 0-4 indicates minimal anxiety, 5-9 as mild anxiety, 10-14 as moderate anxiety, and 15-21 as severe anxiety (as shown in Figure 4 in the Appendices) [18]. Thus, higher scores indicate higher levels of anxiety.

BDI is one of the most widely used self-reported questionnaires and consists of 21 items to measure the severity of depression. Each item is composed of a symptom relating to depression, and participants must select the statement within a multiple-choice format that best describes themselves currently. The items are measured on a 3-point scale, and the score of each item is then combined to calculate the total score, which can range from 0 to 63. A score of 1-10 is considered normal, 11-16 indicates mild mood disturbance, 17-20 as borderline clinical depression, 21-30 as moderate depression, 31-40 as severe depression, and a score greater than 40 as extreme depression [19]. The exact questions and the scoring scale are shown in the Appendices (Figures 5-7). Thus, higher scores indicate higher levels of depression.

Data collection and analysis 

The anonymous data from the survey was collected through REDCap (REDCap, Vanderbilt University, Nashville, USA). Spearman's rank correlation coefficient was used to determine the strength and direction of association of the ordinal data between Mediterranean Diet Adherence Assessment and GAD-7 anxiety assessment, as well as Mediterranean Diet Adherence Assessment and BDI [20].

## Results

Sample

A total of 117 anonymous subjects' responses were initially collected online through RedCap. Seventeen participants were omitted due to the incompletion of the survey. The final sample size consisted of 100 participants between the ages of 19 and 77. The average age was 37 years old with the sample consisting of 61% women and 31% men. Demographics included 79% White, 10% Hispanic, 5% two or more different ethnicities/races, 3% Asian, 2% African American or Black, and 1% Other.

Findings

**Figure 1:**
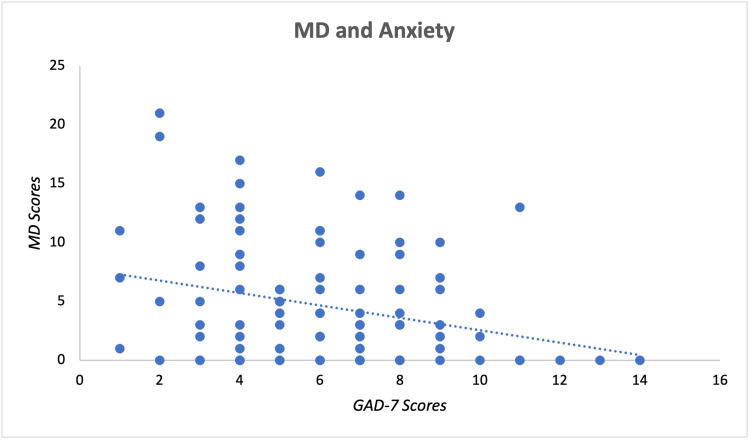
Mediterranean Diet Adherence and Reported Levels of Anxiety Mediterranean diet is represented by MD in the figure. MD scores indicate raw scores obtained from the 14-item questionnaire of Mediterranean Diet Adherence Assessment. GAD-7 scores indicate raw scores obtained from the Generalized Anxiety Disorder-7 questionnaire to assess levels of anxiety. Scores from both questionnaires were plotted together on the appropriate axis for each individual participant and are displayed as a single data point. Raw MD and GAD-7 scores demonstrate a negative relationship, where higher GAD-7 scores are related to lower MD scores.

**Figure 2:**
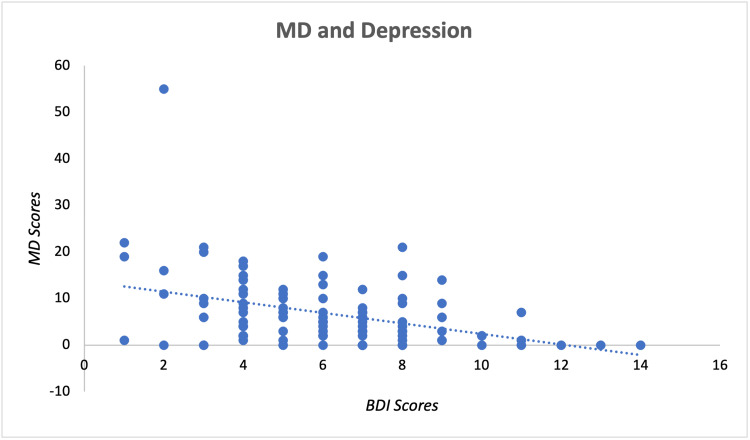
Mediterranean Diet Adherence and Reported Levels of Depression Mediterranean diet is represented by MD in the figure. MD scores indicate raw scores obtained from the 14-item questionnaire of Mediterranean Diet Adherence Assessment. BDI scores indicate raw scores obtained from the Beck's Depression Inventory questionnaire to assess levels of depression. Scores from both questionnaires were plotted together on the appropriate axis for each individual participant and are displayed as a single data point. Raw MD and BDI scores demonstrate a negative relationship, where higher BDI scores are related to lower MD scores.

**Figure 3:**
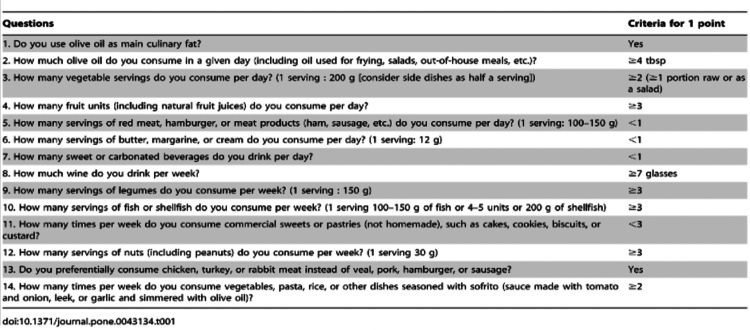
Validated 14-Item Questionnaire of Mediterranean Diet Adherence The 14-item questionnaire used to assess adherence to the Mediterranean diet.

**Figure 4:**
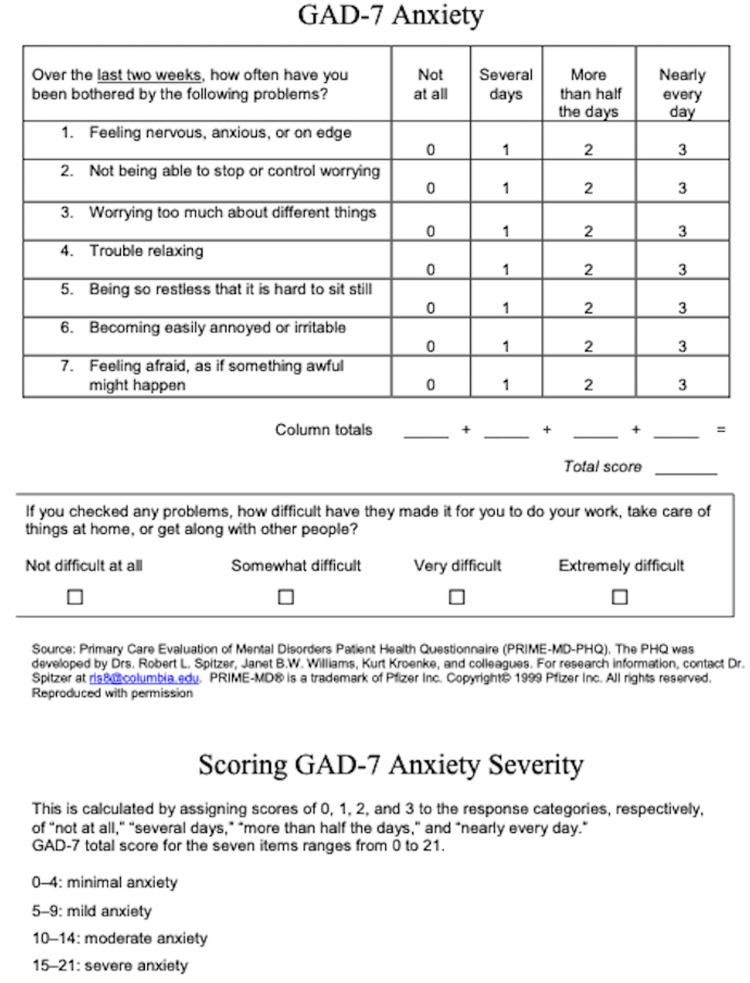
GAD-7 Anxiety Survey to Measure Mental Wellness The Generalized Anxiety Disorder-7 questionnaire to assess levels of anxiety.

**Figure 5:**
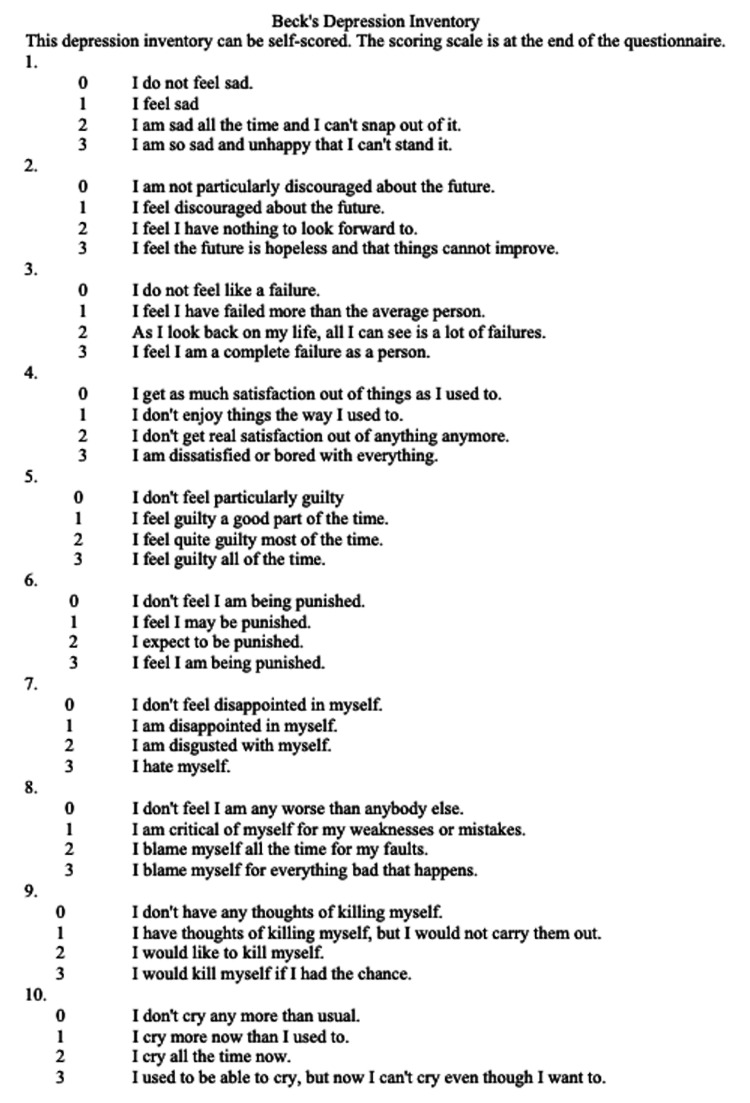
Beck’s Depression Inventory to Measure Mental Wellness First page of the Beck’s Depression Inventory, a 21-item questionnaire to assess levels of depression.

**Figure 6:**
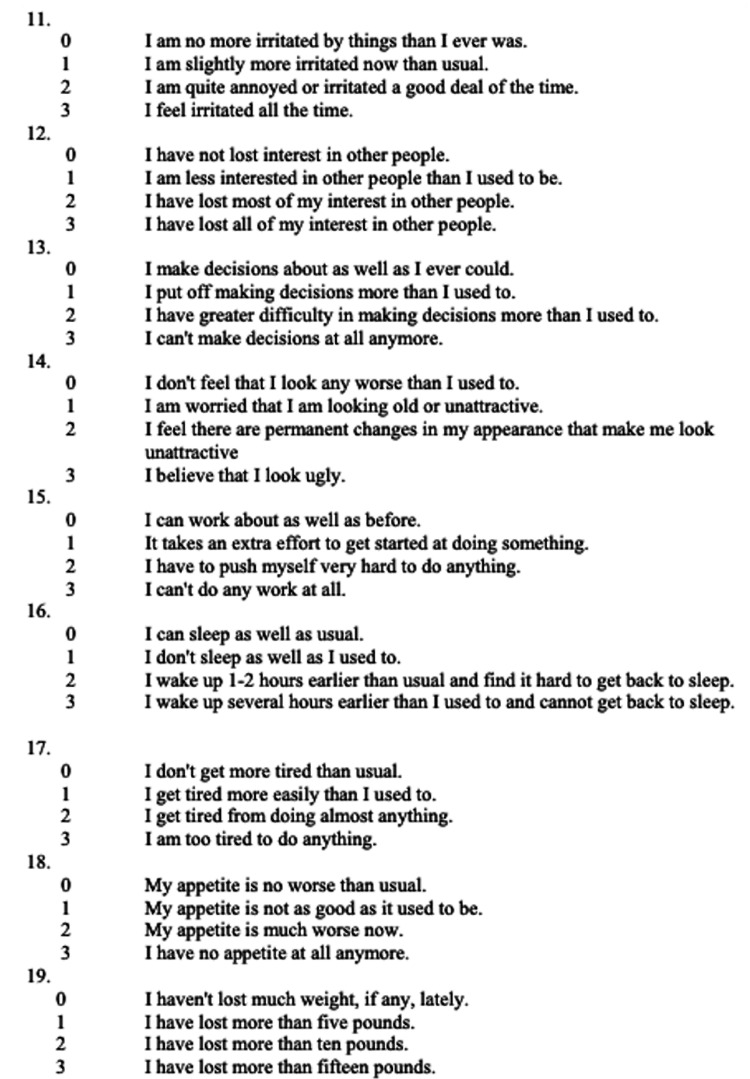
Beck’s Depression Inventory to Measure Mental Wellness Second page of the Beck’s Depression Inventory, a 21-item questionnaire to assess levels of depression.

**Figure 7:**
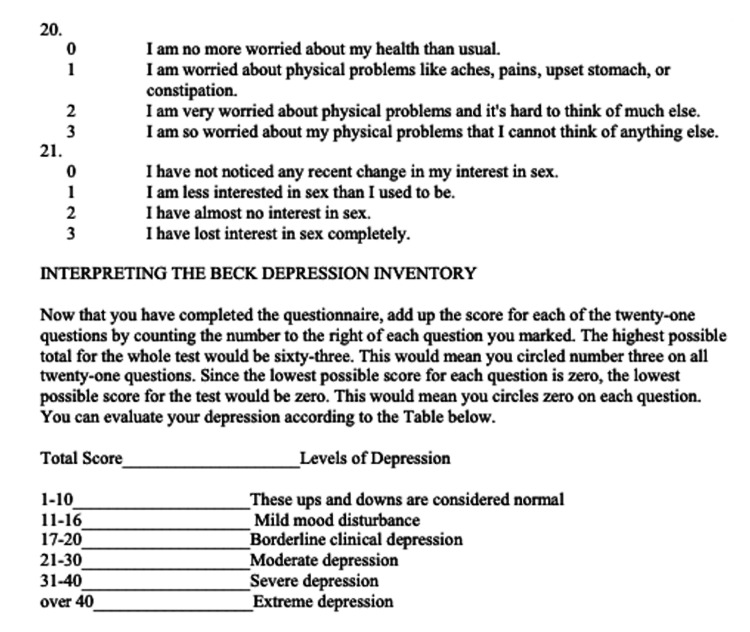
Beck’s Depression Inventory to Measure Mental Wellness Third page of the Beck’s Depression Inventory, a 21-item questionnaire to assess levels of depression.

Scores received by the participants on the 14-item Questionnaire of Mediterranean Diet Adherence [14-17], GAD-7 [18], and BDI [19] were calculated by the researchers according to their specific scoring key created by their original authors. Higher scores for each assessment indicated greater adherence to MD, higher levels of anxiety, and higher levels of depression. The relationship of raw MD and GAD-7 scores is displayed within the scatter plot of Figure 1, while the relationship of raw MD and BDI scores is displayed in Figure 2. Each point in the figures represents an individual participant's scores associated with the designated assessments. Both figures display a general negative relationship, hence higher GAD-7 or BDI scores are related to a lower MD score. 

Spearman’s rank correlation coefficient analysis was further used to investigate our hypothesis and identify any possible correlations between MD and depression as well as MD and anxiety. The raw data of each assessment was initially ranked individually to prepare for data analysis via Spearman’s correlation coefficient (rs). As reported in Table 1, the results of the analysis show Sig. (two-tailed) = 0.0001 for depression and Sig. (two-tailed) = 0.0191 for anxiety. A p-value < 0.05 was selected to assess statistical significance. Therefore, the results indicated that there is a significant correlation between both MD and depression as well as MD and anxiety. Additionally, both depression and anxiety have negative rs values, implying a negative correlation with MD for both. Due to MD and anxiety having an rs value (-0.234) closer to zero compared to MD and depression (rs = -0.369), MD and anxiety demonstrated a weaker association between their ranks contrary to MD and depression having a stronger association. Overall, our results align with our initial hypothesis suggesting that higher adherence to MD is correlated with lower levels of anxiety and depression. 

**Table 1:** Spearman’s correlation analysis Results indicate a significant correlation between both Mediterranean diet and depression as well as Mediterranean diet and anxiety. r_s_ represents Spearman's rank correlation coefficient. N represents number of participants. df represents degrees of freedom. Sig.(two-tailed) represents the two-tailed p-value.

		Depression	Anxiety
Mediterranean Diet	r_s_	-0.369	-0.234
	N	100	100
	T-value	3.93	2.38
	df	98	98
	Sig.(two-tailed)	0.0001	0.0191

## Discussion

The main purpose of our study was to determine if there was a correlation between adherence to the MD and levels of anxiety and depression. Our findings support a negative correlation, indicating that participants who had higher adherence to MD also had lower levels of both anxiety and depression. In general, the results of our study suggest that one’s diet can potentially play a role in one’s overall mental well-being. Prior literature has also supported this by indicating possible bidirectional relationships between diet and potential causative factors such as inflammation and gut-brain axis [21]. 

Additionally, our sample consisted of mainly women with an average age of 37. When examining the demographics in regard to mental health, women appear to be more likely than men to experience both anxiety and depression [6,22,23]. The leading reason for this gender gap is suggested to be due to sex hormones, but the underlying mechanisms still remain unclear [6,24]. In contrast to the average age of 37 within our sample, the National Center for Health Statistics reports that levels of anxiety and depression are most prevalent among those aged 18-29 (21.0%) and least prevalent among those aged 30-44 (16.8%) [22,23]. Fluctuations in women's hormone levels throughout their lifetime such as during puberty, pregnancy, and menopause, can be possibly correlated to those differences in age group prevalence of anxiety and depression [6].

Overall, our findings suggest that expanding the knowledge on the relationship between adherence to MD and anxiety and depression can further influence the field of nutrition and promote the importance of diet and lifestyle changes that can positively impact one’s entire mental well-being. Based on the growing evidence between diet and mood, it is crucial for healthcare professionals to consider diet when evaluating a patient’s lifestyle and well-being. This patient-provider partnership can aid in patients having a deeper understanding of the relationship between diet and mental well-being. Ensuring a proper nutritional state in individuals is known to have an important role in treating mental illness by it improving their emotional and cognitive functioning [25]. Utilizing a food diary in one’s treatment and care plan can be the initial step in implementing this [26].

However, individuals must understand that overall mental well-being and diet are not a “one size fits all.” Some individuals may significantly benefit from MD, while others may not notice any positive effects on their mental state. As more literature is published, individuals need to determine what diet and lifestyle are the most beneficial for them and to discuss this with their healthcare provider for further guidance. Through self-reflection during our study, participants had the opportunity to identify flaws in their diet, which can then be used to identify goals for improvement, thus potentially improving their mental health as well. Ultimately, the results of our study can provide potential evidence regarding the significance of diet in mental well-being. Determining a relationship between one's dietary patterns and overall mental health can encourage and promote public health efforts to improve eating habits. 

Limitations 

The small sample size may prevent the findings from being extrapolated to the overall general public. A convenience sampling resulted in 61% of the participants being women and 79% being White. Since the sample was not significantly diverse, the results of the study may have been skewed and thus results are less likely applied to other ethnic/racial groups. In addition, a voluntary online survey to collect data makes it difficult to know whether participants reported accurately. For example, respondents may select the assumed socially acceptable answer, rather than selecting what they actually felt or consumed on a daily basis. Despite the anonymity, the sensitivity of the questions about anxiety and depression may have caused the participants to feel judged and avoid answering the questions truthfully. This original study provided worthwhile results that validate the positive relationship between dietary quality and mental health. Further studies that incorporate physical activity patterns from participants should be considered due to their confounding potential. Additional questions about participants’ entire well-being can be evaluated, such as existing mental health, medication use, alcohol consumption, tobacco use, and sleep patterns.

## Conclusions

Our study successfully expanded evidence about the negative correlation between MD and levels of anxiety and depression in mental well-being. This association was strongest for depression. Our findings demonstrate the potential therapeutic effect of healthy dietary patterns on mental well-being. The results affirm that patient education and nutrition counseling for lifestyle modifications are essential strategies for healthcare professionals. Future studies to assess comprehensive well-being should be considered to address any possible confounding variables to confirm and strengthen our findings.
